# Public perception of the physician associate profession in the UK: a systematic review

**DOI:** 10.1186/s12913-024-11965-2

**Published:** 2024-11-29

**Authors:** Rhys Swainston, Yingxi Zhao, Eli Harriss, Attakrit Leckcivilize, Mike English, Shobhana Nagraj

**Affiliations:** 1 NDM Centre for Global Health Research Nuffield Department of Medicine, University of Oxford, S Parks Rd, Oxford, OX1 3SY UK; 2 Bodleian Health Care Libraries, University of Oxford, Oxford, UK; 3 KEMRI-Wellcome Trust Research Programme, Nairobi, Kenya; 4 Primary Care Unit, Department of Public Health and Primary Care, University of Cambridge, Cambridge, UK; 5 East London NHS Foundation Trust, London, UK; 6 Nuffield Department of Women’s & Reproductive Health, University of Oxford, Oxford, UK

**Keywords:** Physician associates, Public perception, Patient satisfaction, NHS, Systematic review

## Abstract

**Background:**

The physician associate (PA) role within the NHS is currently under scrutiny due to recent legislative changes and concerns about their scope of practice within primary and secondary healthcare. There is currently limited knowledge of public understanding of PAs and their levels of satisfaction with PAs. This review synthesises the evidence relating to patients’ and potential patients’ understanding of and satisfaction with the PA profession in the UK.

**Methods:**

We systematically searched Ovid MEDLINE, Ovid EMBASE, Ovid PsycINFO, EBSCOhost CINAHL, Education Resources Information Centre (ERIC), ProQuest Dissertations and Theses Global, and Scopus databases for empirical studies of patient understanding of PAs or satisfaction with PAs. We included quantitative, qualitative, and mixed-methods studies looking at PAs in primary and/or secondary care. Quality appraisal was conducted using the CASP Critical Appraisal checklists. A reflexive thematic analysis was used to synthesise data and the GRADE-CERqual method was used to assess the certainty of the themes.

**Results:**

A total of 18 papers involving 15 studies were included in the review. Findings revealed that patients had limited understanding of the PA role with many mistaking PAs for doctors and other healthcare professionals. Patients were confused by the lack of PA prescribing rights. There was receptivity to learn more about the PA role. The evidence suggests that patients had a largely positive view of PAs after an encounter, despite their lack of knowledge about the role. Many patients expressed a willingness to be seen by PAs in future and viewed them as a useful part of the wider healthcare system.

**Conclusion:**

In the UK, there is limited information about, and understanding of, the PA role. Nevertheless, patients were largely satisfied with the quality of care they receive from PAs during consultations. Our findings suggest a need for comprehensive public information regarding the roles and scope of practice of PA’s, and to provide the public and patients with clear expectations of their relative strengths and limitations. Further research might determine if these findings are specific to PAs, or reflect wider issues affecting public perception of other cadres of healthcare professionals.

**Supplementary Information:**

The online version contains supplementary material available at 10.1186/s12913-024-11965-2.

## Background

The NHS is undergoing an unprecedented workforce crisis [1, 2]. Physician Associates (PAs) are healthcare professionals with two years of post-graduate training introduced to the UK in 2003 [3, 4]. They work as part of an interprofessional healthcare team under the supervision of doctors [3–5]. PAs were introduced in the USA in the 1960s [6] and are publicly recognised healthcare professionals, however their role within the UK National Health Service (NHS) remains understudied. There are currently over 3000 PAs employed in the NHS [7], and the recent NHS workforce plan outlines expansion of PA numbers to 10,000 by 2036, alongside 60,000 to 74,000 doctors [3]. PAs are likely to play a larger part in UK healthcare delivery over the coming years; however, their professional role within the NHS has divided the healthcare community [4]. Legislation passed in the House of Commons in February 2024 giving the General Medical Council (GMC) authority to professionally regulate PAs [8] received a mixed response [9–12]. Proponents of PAs pointed to the need for a cadre of healthcare professionals who provide continuity of care and do not rotate as often as junior doctors - PAs are able to familiarise themselves within a specific clinical context and improve their skills within a clinical area over time [8]. Opponents of the legislation argued that the regulation of PAs by the GMC would lead to a blurring of professional boundaries with doctors [10, 11], with concerns about the potential impact on public perception. Patient safety concerns have also been raised and whilst most agree that the PA role requires supervision, debate continues regarding who should provide supervision. It is unclear how senior doctors, who already face significant service pressures, will have the time and resources to provide supportive supervision for both PAs and trainee doctors [11].

Recent media attention around PAs has made this a topic of public concern. Whilst there have been reviews of the role of PAs within the NHS and other healthcare systems [12, 13], there is minimal review evidence on public understanding and satisfaction with the profession. Media coverage of high-profile cases involving PAs [14, 15], suggests a lack of awareness amongst members of the public regarding the roles of PAs in the NHS. A recent English survey by Healthwatch in April 2024, asked 1,914 members of the public whether they understood the difference between doctors and PAs; only 52% agreed or strongly agreed that they did [16]. Due to these gaps in our understanding of public perception of PAs, we conducted a systematic review to better understand patients’ and potential patients’ understanding and satisfaction of the PA role.

This review aimed to synthesise the data regarding public perception of PAs in the UK since their implementation in 2003 [4]. Here, we use the term ‘public’ to refer to patients and potential patients. Our review focused on two research questions:

1. What are patients’ and potential patients’ understanding of the physician associate profession in the UK? Here, understanding is defined as the conceptualisation of the PA role.
2. What are the levels of patient satisfaction with the physician associate profession in the UK? Here, satisfaction is defined as patient contentment with the process of care provided, rather than long-term outcomes. This review is focused on patient satisfaction and is not looking at PA success rates in treating ailments. A patient may be satisfied with their treatment even if their ailment has not lessened or been cured.

## Methods

We conducted our systematic review using PRISMA guidelines (see Appendix D) and published our review protocol on PROSPERO (CRD42024541562). The search terms and strategy were devised by an experienced healthcare librarian [EH]. We searched Ovid MEDLINE, Ovid EMBASE, Ovid PsycINFO, EBSCOhost CINAHL, Education Resources Information Centre (ERIC), ProQuest Dissertations and Theses Global, and Scopus databases. The databases were restricted to articles published between January 2003 (when PAs were introduced to the UK) and the April 2024. We limited the review to the UK because of the recent relevant policy changes to PAs in the NHS and as PAs are a comparatively new profession in the UK. We included studies written in the English language with full-text available. Empirical studies, including observational and interventional studies, those collecting quantitative and qualitative data, and mixed-methods studies were included. Papers that included public and/or patient perception, understanding and awareness, experience, and satisfaction of the PA profession were included. Studies where PAs had contact with patients or the public within primary, secondary, and mental health care settings in the UK were included. In the UK NHS system, primary care refers to patients’ first point of contact with the healthcare system and includes General Practice, community pharmacy, dental and eye health services, while secondary care is defined as hospital and community care that is more specialised and requires referral [17].

Non-empirical study designs and those not meeting the above inclusion criteria were excluded. Studies looking exclusively at healthcare professionals’ views of PAs were also excluded unless they discussed the public or patient perspective of PAs (see Appendix A for the full search strategies). The University of Pittsburgh qualitative search filter for Ovid Medline was used to search Ovid Medline and was translated to search the other six databases [18]. In addition, we conducted citation searches of key papers and hand searching of relevant professional journals. Searches were conducted between April and July 2024.

The title and abstract screening was conducted by one reviewer (RS) and a sample consisting of 20% of papers were reviewed by a second independent reviewer (YZ) to ensure consistency. Papers meeting the above eligibility criteria underwent full-text review by two independent reviewers (RS & YZ). Conflicts were resolved by discussion between the two reviewers first and then with a third if necessary (SN). The title/abstract screening was conducted using RAYYAN software. EndNote software was used for reference management. Data were extracted from included full text papers by the main author (RS) with support from the wider team to ensure consistency, into a data abstraction table to include details of: study setting, study design, participants, and findings relevant to the focus of the research questions. Quality appraisal of all included studies was then conducted using the relevant Critical Appraisal Skills Programme (CASP) surveys (see Appendix B). Data were synthesised inductively using a thematic analysis [19], following established guidance on the conduct of narrative synthesis in systematic reviews [20]. The synthesised data were then assessed for robustness based on quality appraisal of the included studies, so that conclusions could be drawn from the data. Both the primary studies’ methodologies and the trustworthiness of the synthesis itself was assessed during this process. Generated themes were analysed using the GRADE-CERQual approach [21] to assess the confidence of the results (see Appendix C). The wider research team comprising of academics, clinicians, and health economists were involved in discussions of the themes to add further validity to the process of data analysis. None of the team were professionally aligned with PAs or other Medical Associate Professional groups.

## Results

### Study characteristics

**Fig. 1:**
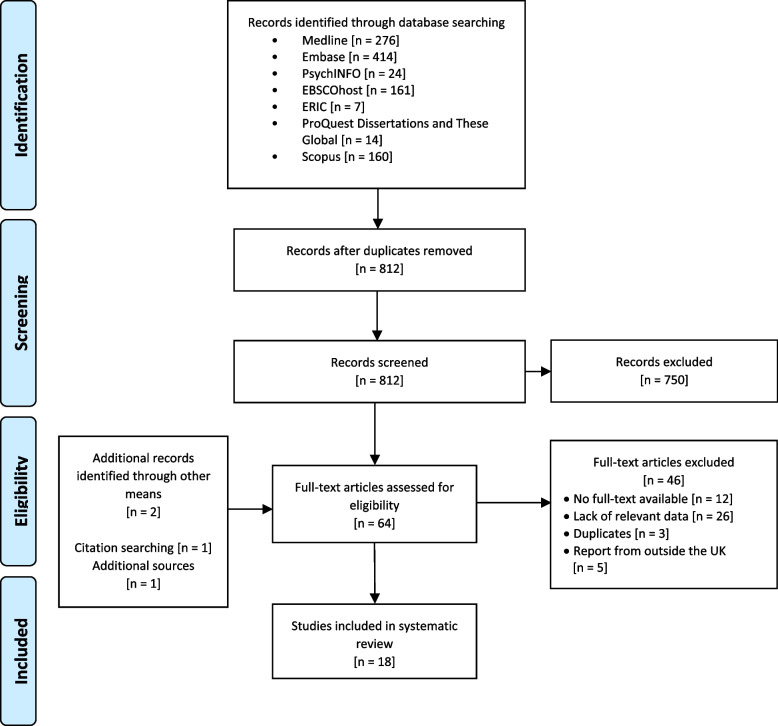
PRISMA flow diagram

A total of 1,056 papers were retrieved in the database search. Of these, 244 were duplicates, leaving 812 papers. The title/abstract review removed a total of 750 papers due to their lack of relevance to this review’s aims, while a further 46 were removed after the full-text review (see Fig. 1). Studies that were excluded from the review typically contained data on PAs (such as competence assessments or healthcare professionals’ perspective) but no data on public or patients’ perspectives. We found an additional full-text published report through citation searching which met the inclusion criteria.

Eighteen papers reporting on fifteen UK studies met the eligibility criteria for inclusion in the review. Only two studies focused primarily on patient perception or experience of PAs, with the majority including data on patient perception as part of a larger aim, typically as part of a general evaluation of PAs (see Table 1 for study characteristics). Three included studies [22–24] reported data collected as part of previous large-scale studies on PAs [25, 26].

**Table 1:** Study characteristics

	Studies	Study Design	Data type	Care Setting	Location	Participants	Data Collection Method	Analysis	Overview of Findings	Quality Appraisal
1	Jackson, Marshall, & Schofield [2017] [27]	Qualitative study	Qualitative	Primary	England	Patients, GPs, ANPs	Focus groups	Thematic analysis	Patients overall lacked understanding of the PA role and expressed confusion over prescribing rights.	High
2	Shah et al. [2021] [28]	Survey design	Quantitative, qualitative	Secondary	England	Patients	Patient feedback survey	Descriptive	Patient-centred survey found views on PAs were very positive.	Low
3	Taylor et al. [2020] [29]	Interpretive methodology	Qualitative	Secondary	England	Patients, PAs	Semi-structured interviews	Thematic Analysis	Tested feasibility of leaflet introducing PAs. Results positive.	High
4	Taylor, Halter, & Drennan [2019] [30]	Qualitative study	Qualitative	Secondary	England	Patients, representatives of patients	Semi-structured interviews	Thematic analysis	Interviews with patients found that they were generally satisfied with PAs but lacked an understanding of the role.	High
5	McDermott et al. [2022] [31]	Mixed-methods	Quantitative, qualitative	Primary	England	Patients	Observations, interviews, focus groups	Thematic analysis	Focus on skill-mix in GP clinics. Experiences with PAs were good.	High
6	Farmer et al. [2011] [32]	Mixed-methods longitudinal study	Quantitative, qualitative	Primary, secondary	Scotland	PAs, healthcare practitioners	Interviews, feedback forms, activity data collection	Thematic analysis	PAs viewed as effective and good communicators.	Moderate
7	Williams & Ritsema [2014] [33]	Survey research	Quantitative	Primary, secondary	UK	Doctors	Survey	Descriptive	Doctors report that patient satisfaction with PAs is high.	High
8	Halter et al. [2020] [34]	Mixed-methods study	Quantitative, qualitative	Secondary	England	Patients, PAs, healthcare professionals	Chart review, observations, semi-structured interviews	Thematic analysis, statistical analysis	Patients were satisfied with PAs but did not know much about them.	High
9	Halter et al. [2017c] [22]	Qualitative study	Qualitative	Primary	England	Patients	Semi-structured interviews, survey	Thematic analysis	Overall, patients were satisfied with PAs but many did not know much about the role	High
10	Cheang et al. [2009] [35]	Cross-sectional survey study	Quantitative	Secondary	England	Patients	Survey	Unknown	20% of participants identified PAs as medically qualified.	Low
11	Zaman et al. [2018b] [36]	Survey study	Quantitative	Secondary	England	Patients	Survey	Statistical analysis	Patients were overall satisfied with PA expertise and quality of care.	High
12	Drennan et al. [2020] [37]	Mixed-methods longitudinal study	Quantitative, qualitative	Secondary	England	Senior clinicians, healthcare professionals	Semi-structured interviews, document analysis	Thematic analysis	PAs perceived to be accepted by patients.	High
13	Drennan et al. [2019] [23]	Mixed-methods case study design	Quantitative, qualitative	Secondary	England	PAs, healthcare professionals, managers	Interviews, observations, work diaries, documentary analysis	Thematic analysis, descriptive analysis	PA’s perceived in a positive light overall.	High
14	Drennan et al., [2019] [PA-SCER] [26]	Mixed-methods multiphase design	Quantitative, qualitative	Secondary	England	PAs, MDs, patients	Systematic review, policy review, national surveys, case studies, interviews, pragmatic retrospective record	Thematic analysis, descriptive analysis, ethnographic vignettes	Patients were found to be positive about PAs but knew little about them.	High
15	Drennan et al. [2015] [24]	Comparative observational study	Quantitative	Primary	England	Patients, PAs, healthcare professionals, management, administration	Observation, survey	Statistical analysis	High reports of satisfaction but no statistically significant difference between PAs and GPs	High
16	Drennan et al. [2014] [25]	Mixed-methods study	Quantitative, qualitative	Primary	England	PAs, patients, nurses, administrative staff	Empirical review, scoping review, surveys, semi-structured interviews, observations	Thematic analysis, descriptive analysis	Majority of patients had positive experiences with PAs.	High
17	Woodin et al. [2005] [38]	Mixed-methods case study design	Quantitative, qualitative	Primary, secondary	England	PAs, patients, practice staff, stakeholders	Interviews, analysis of data records, literature review, focus groups	Realistic evaluation	Patients knew little of PAs but were overall satisfied with their quality of care.	Moderate
18	Wilsher et al. [2023] [39]	Convergent mixed-methods case study design	Quantitative, Qualitative	Secondary	England	PAs, patients/relatives of patients	Survey, semi-structured interviews	Descriptive analysis, thematic analysis	Pass provide effective care in acute hospital settings. Patients had positive views on them.	High

Thirteen studies were ranked as ‘high’ quality, with three ranked as ‘moderate’, and two ranked as ‘low’ using CASP quality appraisal checklists. ‘Low’ ranks were given primarily due to the studies including little to no explanation of analysis in the methods. Over half of the included papers included both quantitative and qualitative data (*n* = 10), with *n* = 4 quantitative studies, and *n* = 4 qualitative studies. There were *n* = 5 studies situated in primary care settings, *n* = 10 in secondary care settings, and *n* = 3 in both. All studies were conducted between 2005 and 2023.

Five major themes were identified from the data. Themes 1 and 2 pertained to the first research question on patient understanding; themes 3–5 pertained to the second research question on patient satisfaction (see Table 2).

**Table 2:** Themes identified

Theme Table		
Theme	**Sub-theme**	**Supporting Data**
1. Patients lacking information about PAs	Unfamiliarity with the PA role	*“None of the patients or relatives who were interviewed mad met a PA prior to this hospital episode. None of them had an accurate understanding of what a PA was.” (Drennan et al.*,* 2019* [26],* page 50)*
	Information provided about PAs	*“The letter didn’t explain that… maybe that needs to be explained there is a new role. I mean it’s not to bother any people*,* it’s just to give some more understanding.” (patient*,* Taylor*,* Halter*,* & Drennan*,* 2019*, [30], * page 7)*
	Confusion regarding prescribing rights of PAs	*“However, there was much confusion about the ability of an advanced nurse practitioner to sign prescriptions when a PA, seen by most patients as more highly trained, could not.” (Woodin et al, 2005* [38],* page 103).*
2. PAs mistaken for other healthcare workers	PAs mistaken for doctors	*“I called him doctor because I thought he was a doctor . . . I don’t know why they didn’t tell me, I’m not sure whether they didn’t want me to think he wasn’t a doctor and to think that he wasn’t going to do such a good job…” (Drennan et al*,* 2014* [25],* page 73)*
	PAs mistaken for non-physician healthcare workers	*“Some participants offered accounts of mistaken identity and described thinking that the PA was variously a junior doctor in training, a ‘floating doctor, ‘like a supply doctor’, a senior nurse or ‘a nice member of staff in the clerking department’.” (Drennan et al, 2019* [26],* page 50)*
3. Patients’ experiences being seen by a PA	PA competency	*“My experiences have been very good. In fact, with the physician associate, I found her very good because I have a… quite a lot of ailments, a lot of things wrong with me, and she’s more understanding of looking at me as a whole rather than just dealing with an isolated ailment… And I find that she’s been very, very good at dealing with everything and joining the dots up a little bit.” (McDermott et al, 2022* [31],* page 61)*
	PA attitude and behaviour	*“PAs were variously described as “professional”*,* “confident” and “calm”; these positive mannerisms were attributed by participants to enhanced confidence in the PA’s skills and knowledge.” (Taylor*,* Halter*,* & Drennan*,* 2019* [30],* page 4)*
4. Patient willingness to be seen by a PA	Patient willingness to be seen by a PA	*Patient survey results*: *“I would be happy to see a PA again: 100% of patients replied with strongly agree/agree.” (Zaman et al.*,* 2018b* [36],* page 215)*
5. Patients views on the healthcare system	Patient trust in the healthcare system	*“Participants also expressed trust in the PA derived from their trust and confidence in their general practice*,* particularly the senior partners*,* and also in the wider system of the NHS.” (Drennan et al*,* 2014* [25],* page 75)*
	Patients’ perception of the PA role within healthcare	*“Most of our patient participants were receptive to the role on the grounds that it might speed up care.” (Halter et al.*,* 2020* [34],* page 7)*

### Limited understanding of the PA role

#### Unfamiliarity with the PA role

Participants across both primary and secondary care reported being unfamiliar with the PA role [26, 27, 29, 30, 33, 38, 39], with many patients reporting limited or incorrect information about them [26, 38]. Patients’ reported descriptions of PAs were typically inaccurate [38]; one participant described PAs as “trainee physicians” [22]. Researchers often described patients as ‘confused’, and ‘unaware’ regarding PAs [30, 38]. Few participants reported being seen by a PA in the past [25] and researchers were sometimes the first to point out to patients in the studies that the healthcare professional they had seen was a PA [26, 30]. For many, the title of ‘PA’ itself was new:

*“None of the patients or relatives who were interviewed had met a PA prior to this hospital episode. None of them had an accurate understanding of what a PA was (Drennan et al., 2019 [* 26 *], page 50)”*

One study [39] surveyed patients following treatment by PAs; all but one stated they did not know about the PA role before their stay in hospital. Another noted that only 20% of 190 ear, nose, throat (ENT) patients understood that PAs were medically qualified [35].

#### Information provided about PAs

Patients across studies wanted more information about PAs [29, 30, 38]. In one study, patients expressed an interest in better understanding PAs’ role within healthcare, their qualifications, and the distinction between PAs and other healthcare roles [38]. Patients expressed confusion as to why this information wasn’t provided in the first place:

*“The letter didn’t explain that. maybe that needs to be explained there is a new role. I mean it’s not to bother any people*,* it’s just to give some more understanding.” (patient*,* Taylor*,* Halter*,* & Drennan*,* 2019* [30], *page 70)*

Patients were receptive to information provided about the PA role. This is evidenced by a study by Taylor et al., 2020 [29] who designed and evaluated a patient information leaflet explaining the PA role in secondary care. The study noted that patients found the leaflet intervention acceptable, though some wanted further information on the role. A small number of studies noted that PAs provided information about their role to patients, only for it to be ignored or forgotten [25, 29]. One study noted that secondary care patients rarely questioned PAs on their role in healthcare [39]. Patients across studies stated they had little interest in who they were being seen by, with some remarking that they were just happy to be seen at all [26, 29, 38].

#### Confusion regarding prescribing rights of PAs

Several studies noted that patients were perplexed by PAs’ lack of prescribing rights [25, 27, 38, 39]. Comments were made pertaining to the disconnect between a PAs’ qualifications, and their ability to prescribe:

*“However*,* there was much confusion about the ability of an advanced nurse practitioner to sign prescriptions when a PA*,* seen by most patients as more highly trained*,* could not” (Woodin et al.*,* 2005* [38], *page 103)*

The lack of prescribing rights occasionally caused frustration [25]; most patients, however, were largely unbothered by the delays it caused by the need for supervision or referral to a doctor for signing prescriptions [25, 38]. One study noted that the PA checking with a doctor reassured patients that the prescription was correct [38]. Studies also reported that participants thought the lack of prescribing rights could cause problems for PAs, stating that others could perceive them as less qualified [27, 38].

### PAs mistaken for other healthcare professionals

#### PAs mistaken for doctors

A common theme across multiple studies related to patients perceiving a PA they had seen as being doctors [26, 29, 30, 32–34, 39]. Most of this confusion was found in studies based in secondary care [23, 25, 26, 29, 30, 33, 34, 38, 39]. One study also noted PAs and doctors reporting this confusion in their patients [33]. Reasons listed for the confusion included the PAs’ attire, manner of speaking, and the type of medical procedure being conducted [30]. Patients’ lack of understanding of the PA role was exemplified by the number of instances of PAs being mistaken for doctors:

*“I called him doctor because I thought he was a doctor. I don’t know why they didn’t tell me*,* I’m not sure whether they didn’t want me to think he wasn’t a doctor and to think that he wasn’t going to do such a good job…” (patient*,* Drennan et al.*,* 2014* [25], *page 73)*

Patients across studies stated that they were given no indication that they were being seen by a PA, with many simply assuming they were being seen by a doctor [25, 26, 39]. This implies that there were limitations in the amount and quality of information provided to patients about their consultation.

#### Patients mistaking PAs for non-physician healthcare workers

Though less frequent, patients also reported confusing PAs with non-physician healthcare professionals in both primary [25] and secondary [26] care:

*“Some participants offered accounts of mistaken identity and described thinking that the PA was variously a junior doctor in training*,* a ‘floating doctor*,* ‘like a supply doctor’*,* a senior nurse or ‘a nice member of staff in the clerking department’.” (Drennan et al.*,* 2019* [26], *page 50)*

Patients perceived PAs to be nurses or nurse practitioners [25]; however, one participant stated they thought the PA might be an administrative assistant [26].

### Patient experiences being seen by a PA

#### Competency of PAs

Across both primary and secondary care, PAs were described by patients as competent, effective, and efficient [30, 32, 36, 37], as well as being confident and professional [25, 38]. Many patients openly stated their trust in PA expertise [26, 37]. Some patients expressed that their PA had consulted with a doctor, something the patients viewed as a sign of clinical competence [25, 26]. Referring to a doctor was viewed as an example of the PA doing their job properly, alongside professionalism, confidence, and efficiency:

*“My experiences have been very good. In fact*,* with the physician associate*,* I found her very good because I have a… quite a lot of ailments*,* a lot of things wrong with me*,* and she’s more understanding of looking at me as a whole rather than just dealing with an isolated ailment… And I find that she’s been very*,* very good at dealing with everything and joining the dots up a little bit.” (patient*,* McDermott et al.*,* 2022* [31], *page 61)*

One study gathered quantitative survey data on 86 patients’ views on PAs, finding that 97% were overall satisfied with the quality of care they received [36]. A small number of participants also compared PAs favourably with other healthcare professionals, particularly doctors [25, 31, 38]. PAs were described as more informal and easy-going than doctors and that they spent lots of time explaining things to the patient [31, 38]. In one instance, PAs were also compared favourably to advanced nurse practitioners [38].

Though patients’ comments were mostly positive, some negative comments were also made [25, 30]. Most comments focused on not trusting the PA [25] and feeling as though the consultation was rushed:

*“I felt it was very much on the surface and I came away*,* and they gave me this form and I thought at that time*,* I wasn’t happy with that*,* it didn’t work for me. I was disappointed with the non-outcome of that visit.” (patient*,* Drennan et al.*,* 2014* [25], *page 75)*

For some patients, PAs’ consulting with their supervising doctors was seen as a limitation of their role, rather than a sign of competence and safe practice [25]. Drennan et al. (2014) noted patient dissatisfaction with the extended waiting times caused by the need for supervision [25]. It also noted PAs’ lack of prescribing rights as a problem for some patients. Overall, however, patients viewed PAs as skilled and highly effective across several studies [25, 26, 30, 32, 36, 37].

#### PA attitude and behaviour

As well as being viewed as skilled, PAs were often considered polite and well-mannered to patients, with good communication skills, patience and a personable attitude [24–26, 29, 30, 38, 39]:

*“PAs were variously described as “professional”*,* “confident” and “calm”; these positive mannerisms were attributed by participants to enhanced confidence in the PA’s skills and knowledge.” (Taylor*,* Halter*,* & Drennan*,* 2019* [30], *page 4)*

Communication was consistently highlighted as a skill [25, 26, 28, 30, 38, 39]. PAs were frequently described as being good listeners, and explaining answers to queries in a way the patient could understand [28]. They were also viewed as accessible, with one non-native English-speaking participant stating that the PA had adapted their language to ensure the patient understood [30]. Several survey studies found high levels of patient satisfaction with PA attitudes and communication styles [24, 28, 33, 36, 39]. Some studies found that patients were able to develop good relationships with PAs [25, 30]; they were described as taking a more personable approach to healthcare and were considered reassuring and polite [26, 28]. PAs were again compared favourably to doctors, with patients in one study describing them as better communicators in comparison [26]. Comparatively few negative comments were made pertaining to PA attitude and behaviour [30], showing that overall, patients across studies were found to be satisfied both with PAs’ expertise and attitude/behaviour. The majority of studies that highlighted PA attitude and behaviour were based in secondary care facilities [26, 28–30, 37–39].

### Patient willingness to be seen by a PA

Studies reported high levels of patient willingness to be seen by a PA across both primary [25, 31, 38] and secondary care [30, 36–39], particularly those who had been seen by one recently and had a positive experience [25, 38]. Many also stated they would recommend their friends and family members seek a PA in future [30, 36]. Many participants stated that, if given the choice, they would choose to be seen by a PA:

*“Now if I had a choice I would ask for (the PA) above the other (GP) partners…” [patient*,* Woodin*,* 2005* [38], *page 104]*.

One study found that patients made spontaneous comparisons between PAs and doctors, with PAs being seen as less ‘hierarchical’ and easier to pose questions to [30]. One patient also expressed a preference for seeing a PA over seeing a doctor or GP due to the PA, looking at her symptoms as a whole, unlike GPs [31]. A quantitative study found that 91% of participants (*n* = 86) would recommend being seen by a PA [36]; another found that thirteen out of fourteen participants were happy to be seen by a PA in future [39]. Again, communication was seen as a strength of PAs [25, 30]. However, some participants stated a preference for seeing a doctor [25, 30, 38]. Others expressed apathy regarding who they would like to be seen by, with some patients noting that they were happy so long as they were seen by a competent professional [26, 29, 30].

### Patients views on the healthcare system

#### Patients’ perception of the PA role within healthcare

Though less prevalent than other themes, patients’ views on the healthcare system itself (that is, their views on healthcare practice and the role PAs play in the system) was also of note [25–27, 30, 34, 38]. PAs were largely viewed as contributing to healthcare by providing continuity to patient care and support to other healthcare workers:

*“So the thought that there is a role within the surgery where I could go and see somebody who isn’t as pressurised as the doctor*,* .is a really good thing to have in the surgery and I feel that I would be happy to utilise that again*,* definitely.” (patient*,* Halter et al.*,* 2017c* [22], *page 1016)*

Patients also noted shorter waiting times and described PAs as being a ‘relief’ for healthcare [26]. PAs were also observed by patients to work well within the context of the primary healthcare team [38].

#### Patient trust in the healthcare system

Patients’ confidence in PA was found to partially stem from their trust in the healthcare system generally [25, 30]. Patients reported that their confidence in a PA’s decision was due to agreement from a doctor [30]. Patients also reported trusting the NHS, stating that if the system had placed PAs in that role, then it must be the right role for them [25].

## Discussion

This systematic review gathered and synthesised data on patients’ and potential patients’ perspectives on PAs in the NHS from 2003, when PAs were introduced in the UK. Data synthesis found that overall, patients had little to no awareness or understanding of the PA role. PAs were often mistaken for doctors or for other healthcare professionals. Many patients were confused by PAs’ lack of prescribing rights and some expressed a preference for being seen by a doctor, though this was a minority. Despite patients’ overall lack of awareness, PAs were viewed by them as confident and capable by patients with good attitudes and communication skills. Some studies noted that participants had faith in the NHS to place staff in appropriate positions and PAs were seen as an asset to the healthcare system. Our findings are supported by both the aforementioned Healthwatch survey [16] and the recent BMA survey [40] which found that patients largely did not understand the PA role. Another survey of 1,100 British adults by Ipsos in 2024 similarly revealed that public knowledge of PAs was minimal [41]. However, it noted that 40% of participants wanted the NHS to train more PAs in order to reduce waiting times [41]. Our findings are also supported by a recent study by King et al. (2024), which noted a majority of patient participants did not understand the PA role but that 84% rated their quality of care by a PA as “excellent” [42]. Furthermore, a 2022 qualitative thesis which looked at patient understanding of and satisfaction with PAs in Wales found that patients were unfamiliar with PAs and wanted more information on them, but were nevertheless satisfied with the care they had received [43].

A constant theme across the majority of studies was patients mistaking PAs for doctors. This seems to be tied directly to another finding of our review, that patients in the NHS are poorly informed of the PA role. This lack of information would certainly explain the confusion and many of the studies in our review noted, that patients actively wanted more information on PAs. It is important, however, that other explanations behind this misidentification are considered. For example, studies show that gender plays a role in patients misidentifying healthcare staff in both the US and UK, with female doctors regularly mistaken for nursing staff by patients [44–46]. It is possible that some of the misidentification of PAs by patients could be explained by gender assumptions rather than confusion surrounding the PA role, with female PAs being mistaken for nurses and male PAs being assumed to be doctors. As PAs in England are primarily female [47], further research should be conducted to see what role (if any) gender plays on PA role misidentification.

Research into role recognition also shows that patients have a difficult time correctly identifying their healthcare providers’ role within the healthcare system generally [48]. It is possible, therefore, that patient misidentification is not specific to PAs and that the results of this review do not necessarily support the argument that hiring PAs blurs professional boundaries [11, 12]. This is certainly plausible given the variety of healthcare roles present in both primary and secondary care, including junior doctors, nurses, nurse practitioners and other allied health workers, all of which could add to the confusion. This can be seen in our data, as patients mistook PAs for a variety of roles, including doctors, nurses, or even administrative staff [25, 26, 29, 30, 33, 38]. In future, research into PA misidentification should take into account gender bias and compare the levels of misidentification with other healthcare roles.

Despite this consistent misidentification, patients made many positive comments about PAs, much of them directed at their manner, personable approach, and communication skills. Positive comparisons made with other healthcare staff concerned their personability and attitude rather than their medical skill. This suggests that patients highly value healthcare workers’ demeanour and approach. This is supported by previous research into patient-doctor relationships; Merriel et al.’s 2015 study found that deep patient-doctor relationships led to longer consultations with more in-depth discussion of symptoms [49]. This supports the idea that patients are more concerned with the relationship and rapport they can build with a healthcare practitioner, and the timeliness of their treatment than with what title the practitioner holds. This is not universal, however, as this review found some instances of patients stating a preference for seeing doctors.

### Strengths and Limitations

This review is important and timely, as there currently exists some controversy surrounding the deployment of PAs in the NHS and recent legislation changes are coming under scrutiny. One limitation is that twelve non-full-text conference abstracts were excluded from the review. This was done as the abstracts did not meet the inclusion criteria as without the full texts a quality appraisal of the studies could not be conducted. Attempts were made to contact the authors of the abstracts to ask if full text papers were available, however these attempts were unsuccessful. As this study is focused exclusively on the NHS, we understand that public perception of PAs may vary in different contexts. We acknowledge that the current climate around PAs in the UK is rapidly changing. We also limited our searches to published peer review papers, with surveys from professional agencies such as the British Medical Association [40] and Healthwatch [16] excluded. These surveys have however, been presented in our discussion as part of the wider literature. The analysis was led by one reviewer, however to ensure validity of findings, a second independent reviewer screened the full-text review to determine which papers were eligible for inclusion. Findings were discussed at regular weekly meetings with all authors as part of the data synthesis process. We conducted a narrative synthesis of our review findings and did not synthesise the qualitative and quantitative data separately. This was due to the small number of eligible papers that involved quantitative data, and qualitative synthesis was determined to be most appropriate method for answering our research questions.

## Conclusion

The principal findings of this review are that NHS patients have limited information and understanding of the PA role, but are nevertheless largely satisfied with the quality of care they receive from PAs. The review has gathered important data given recent legislative changes and plans to expand PA numbers outlined in the NHS Workforce plan. Our findings suggest there is a need for comprehensive information regarding definition of the PA role, their scope of practice and to provide the public and patients with clear expectations of their strengths and limitations, their qualifications and role within the wider healthcare team. Future research might focus on public perception of the PA role in the wider context of trust and professional identities within the health system, the role of gender, and comparison with the international literature, to discover if the issues highlighted in this review are specific to PAs in the NHS, or more general to other cadres of health professionals both in the UK and across a variety of contexts.

## Supplementary Information

Supplementary Material 1.

Supplementary Material 2.

Supplementary Material 3.

Supplementary Material 4.

## Data Availability

All data relevant to the study are included in the article or uploaded as tables and supplementary materials.

## References

[1] Cooksley T, Clarke S, Dean J, Hawthorne K, James A, Tzortziou-Brown V, et al. NHS crisis: rebuilding the NHS needs urgent action. BMJ. 2023;380:1. 36596578 10.1136/bmj.p1

[2] Ireland B. MPs Highlight ‘Greatest Workforce Crisis in History’ of NHS. In: BMA. 2022. https://www.bma.org.uk/news-and-opinion/mps-highlight-greatest-workforce-crisis-in-history-of-nhs#:~:text=The%20English%20NHS%20is%20in,health%20and%20social%20care%20committee. Accessed 8 Aug 2024.

[3] NHS England. NHS Long Term Workforce Plan [Internet]. In: England NHS. 2023. https://www.england.nhs.uk/long-read/nhs-long-term-workforce-plan-2. Accessed 8 Aug 2024.

[4] Bagenal J. Physician associates in the UK and the role of the doctor. Lancet. 2024;13:102–4. 10.1016/S0140-6736(24)01401-6 38972322

[5] Wang H, English M, Chakma S, Namedre M, Hill E, Nagraj S. The roles of physician associates and advanced nurse practitioners in the National Health Service in the UK: a scoping review and narrative synthesis. Hum Resour Health. 2022;20:69. 10.1186/s12960-022-00766-5 PMC9479410 36109746

[6] AAPA. History of the PA Profession and the American Academy of PAs. In: AAPA.org. 2016. https://www.aapa.org/about/history/. Accessed 8 Aug 2024.

[7] BMA. Medical associate professions (MAPs) [Internet]. 2024 [cited 8 August 2024]. Available from: https://www.bma.org.uk/advice-and-support/nhs-delivery-and-workforce/workforce/medical-associate-professions-mapsBMA.

[8] Hansard, Anaesthesia Associates And Physician Associates Order. 2024. In: UK Parliament. 2024. https://hansard.parliament.uk/Lords/2024-02-26/debates/4ED09D68-187C-4325-B4F3-E9F23712FD0C/AnaesthesiaAssociatesAndPhysicianAssociatesOrder2024. Accessed July 9 2024.

[9] Ghadiri SJ. Physician associates: an asset for physician training and a 21st-century NHS? Future Healthc J. 2020;73:e9–10. 10.7861/fhj.teale-7-3 PMC7571733 33094238

[10] Clarke S. There is a role for physician associates in the NHS. BMJ. 2024;384:q618. 38471731 10.1136/bmj.q618

[11] Salisbury H. Helen Salisbury: Physician associates in general practice. BMJ. 2023;382:1596. 10.1136/bmj.p1596 37433612

[12] Salisbury H. Helen Salisbury: blurring the boundaries of the medical profession. BMJ. 2024;384:q494–4. 10.1136/bmj.q494 38418100

[13] Zhao Y, Quadros W, Shobhana N, Wong G, English M, Leckcivilize A. Factors influencing the development, recruitment, integration, retention and career development of advanced practice providers in hospital health care teams: a scoping review. BMC Med. 2024;22(1):286. 10.1186/s12916-024-03509-6 PMC11232288 38978070

[14] Ennals E. Newborn left disabled after signs of serious illness were missed by physician associate. In: The Mail Online. 2024. https://www.dailymail.co.uk/health/article-13679799/Newborn-left-disabled-signs-illness-missed-physician-associate.html. Accessed on 13 Aug 2024.

[15] Ungoed-Thomas J. Wider use of physician associates will increase inequality, say UK doctors. In: The Observer. 2024. https://www.theguardian.com/society/article/2024/jun/30/wider-use-of-physician-associates-will-increase-inequality-say-uk-doctors. Accessed 18 Jul 2024.

[16] Healthwatch. Am I seeing a physician associate or a doctor? In; Healthwatch.com. 2024. www.healthwatch.co.uk. 2024 https://www.healthwatch.co.uk/blog/2024-07-22/am-i-seeing-physician-associate-or-doctor. Accessed 2 Aug 2024.

[17] NHS Digital. The healthcare ecosystem. NHS Digital. 2022. https://digital.nhs.uk/developer/guides-and-documentation/introduction-to-healthcare-technology/the-healthcare-ecosystem.

[18] University of Pittsburgh. Ovid Medline search filters: qualitative studies (revised 25 March 2024) 2024 [updated 25/03/2024]. Accessed 25 July 2024.

[19] Braun V, Clarke V. Thematic analysis: a practical guide. London: SAGE; 2022.

[20] Popay J, Roberts H, Sowden A, Petticrew M, Arai L, Rodgers M, et al. Guidance on the Conduct of Narrative Synthesis in systematic reviews a product from the ESRC Methods Programme. Lancaster: Lancaster University; 2006.

[21] Cerqual.org. GRADE-CERQual-Home. In: Cerqual. 2024. https://www.cerqual.org. Accessed on 9 July 2024.

[22] Halter M, Drennan VM, Joly LM, Gabe J, Gage H, de Lusignan S. Patients’ experiences of consultations with physician associates in primary care in England: a qualitative study. Health Expect. 2017;205:1011–9. 10.1111/hex.12542 PMC5600217 28429886

[23] Drennan VM, Halter M, Wheeler C, Nice L, Brearley S, Ennis J, et al. What is the contribution of physician associates in hospital care in England? A mixed-methods, multiple case study. BMJ Open. 2019;9(1):e027012. 10.1136/bmjopen-2018-027012 PMC6359738 30700491

[24] Drennan VM, Halter M, Joly L, Gage H, Grant RL, Gabe J et al. Physician associates and GPs in primary care: a comparison. Br J Gen Pract. 2015;65(634):e344–50. Cited 2020 Jan 17. 10.3399/bjgp15X684877 PMC4408498 25918339

[25] Drennan VM, Halter M, Brearley S, Carneiro W, Gabe J, Gage H, et al. Investigating the contribution of physician assistants to primary care in England: a mixed-methods study. Health Serv Delivery Res. 2014;216:1–136. 25642506

[26] Drennan VM, Halter M, Wheeler C, Nice L, Brearley S, Ennis J, et al. The role of Physician Associates in secondary care: the PA-SCER mixed-methods study. Health Serv Delivery Res. 2019;719:1–158. 31162917

[27] Jackson B, Marshall M, Schofield S. Barriers and facilitators to integration of physician associates into the general practice workforce: a grounded theory approach. Br J Gen Pract. 2017;67(664):e785–91. Cited 2019 Dec 21. 10.3399/bjgp17X693113 PMC5647922 28993304

[28] Shah C, Singh P, Matin S, Farrow J, Magon R, Zia A et al. A physician associate-led clinic for people with severe mental illness in the United Kingdom. JAAPA. 2021;34(8):1–6. Cited 2022 May 2. 10.1097/01.JAA.0000758220.38067.49 34320547

[29] Taylor F, Ogidi J, Chauhan R, Ladva Z, Brearley S, Drennan VM. Introducing physician associates to hospital patients: development and feasibility testing of a patient experience-based intervention. Health Expect. 2020;241:77–86. 10.1111/hex.13149 PMC7879547 33238078

[30] Taylor F, Halter M, Drennan VM. Understanding patients’ satisfaction with physician assistant/associate encounters through communication experiences: a qualitative study in acute hospitals in England. BMC Health Serv Res. 2019;19(1):603. 10.1186/s12913-019-4410-9 PMC6712610 31455342

[31] McDermott I, Spooner S, Goff M, Gibson J, Dalgarno E, Francetic I, et al. Scale, scope and impact of skill mix change in primary care in England: a mixed-methods study. Health Social Care Delivery Res. 2022;109:1–148. 35593786

[32] Farmer J, Currie M, Hyman J, West C, Arnott N. Evaluation of physician assistants in National Health Service Scotland. Scot Med J. 2011;563:130–4. 10.1258/smj.2011.011109 21873716

[33] Williams LE, Ritsema TS. Satisfaction of doctors with the role of physician associates. Clin Med. 2014;142:113–6. 10.7861/clinmedicine.14-2-113 PMC4953279 24715119

[34] Halter M, Drennan V, Wang C, Wheeler C, Gage H, Nice L et al. Comparing physician associates and foundation year two doctors-in-training undertaking emergency medicine consultations in England: a mixed-methods study of processes and outcomes. BMJ Open. 2020;10(9):e037557. 10.1136/bmjopen-2020-037557 PMC7467515 32873677

[35] Cheang PP, et al. What is in a name–patients’ view of the involvement of ‘care practitioners’ in their operations. J Royal Colleges Surg Edinb Irel. 2009;7(6):340–4. 10.1016/s1479-666x(09)80107-8 20681376

[36] Zaman Q, Yogamoorthy S, Zaman M, Fouda RMFR. Patients’ perspective of physician associates in an acute medical unit within an English district general teaching hospital – a pilot survey study. Future Healthcare J. 2018;5(3):213–7. Cited 2020 Apr 13. 10.7861/futurehosp.5-3-213 PMC6502598 31098569

[37] Drennan VM, Calestani M, Taylor F, Halter M, Levenson R. Perceived impact on efficiency and safety of experienced American physician assistants/associates in acute hospital care in England: findings from a multi-site case organisational study. JRSM Open. 2020;11(10):205427042096957. 10.1177/2054270420969572 PMC7705788 33294201

[38] Woodin J, Mcleod H, Mcmanus R, Jelphs K. Evaluation of US-trained Physician Assistants working in the NHS in England. The introduction of US-trained Physician Assistants to Primary Care and Accident and Emergency departments in Sandwell and Birmingham Final report. 2005. https://citeseerx.ist.psu.edu/document?repid=rep1&type=pdf&doi=01cd54c708ca6b371a2a9ee11f30039c41774849. Accessed 9 July 2024.

[39] Wilsher SH, Gibbs A, Reed J, Baker R, Lindqvist S. Patient care, integration and collaboration of physician associates in multiprofessional teams: a mixed methods study. Nurs Open. 2023;10(6):3962–72. 10.1002/nop2.1655 PMC10170911 36808483

[40] BMA. BMA Medical Associate Professions (MAPs) survey Background to the survey. In: BMA. 2024. https://www.bma.org.uk/media/py5h43hp/bma-maps-survey-1.pdf. Accessed 2 Aug 2024.

[41] Ipsos. Ipsos Physician Associates polling. In Ipsos. 2024. https://www.ipsos.com/sites/default/files/ct/news/documents/2024-08/ipsos-physician-associates-polling-july-2024-charts.pdf. Accessed 22 Aug 2024.

[42] King NMA, Helps S, Ong YG, Walker S. Doctors’, patients’ and Physician associates’ perceptions of the Physician Associate Role in the Emergency Department. Health Expectations: Int J Public Participation Health Care Health Policy. 2024;27(4):e14135. 10.1111/hex.14135 PMC11233990 38984378

[43] Morris F. Physician Associates in NHS Wales: A study of the transition from student to qualified clinician, their contribution to teams and services, and responses to the role. In: Orca. 2022. https://orca.cardiff.ac.uk/id/eprint/162495/1/1967970%20Felicity%20Morris%20thesis%20FINAL%20signatures%20removed.pdf. Accessed 9 July 2024.

[44] ‌Berwick S, Calev H, Matthews A, Mukhopadhyay A, Poole B, Talan J, et al. Mistaken identity: frequency and effects of gender-based Professional Misidentification of Resident Physicians. Acad Med. 2021;966:869–75. 10.1097/ACM.0000000000004060 33735130

[45] Ahmad SR, Ahmad TR, Balasubramanian V, Facente S, Kin C, Girod S. Are you really the doctor? Physician experiences with gendered microaggressions from patients. J Women’s Health. 2022;31(4):521–32. 10.1089/jwh.2021.0169 34747651

[46] Jefferson L, Bloor K, Spilsbury K. Exploring gender differences in the working lives of UK Hospital consultants. J R Soc Med. 2015;1085:184–91. 10.1177/0141076814558523 PMC4484206 25567767

[47] Roberts K, Drennan VM, Watkins J. Physician associate graduates in England: a cross-sectional survey of work careers. Future Healthc J. 2022;9(1):5–10. 35372771 10.7861/fhj.2021-0184 PMC8966803

[48] Windish DM, Olson DP. Association of Patient Recognition of Inpatient Physicians with knowledge and satisfaction. J Healthc Qual. 2011;333:44–9. 10.1111/j.1945-1474.2010.00123.x 22414019

[49] Merriel SWD, Salisbury C, Metcalfe C, Ridd M. Depth of the patient–doctor relationship and content of general practice consultations: cross-sectional study. Br J Gen Pract. 2015;65637:e545–51. 10.3399/bjgp15X686125 PMC4513743 26212851

