# Supplementary material for: Public perception of the physician associate profession in the UK: a systematic review
Source: BMC Health Serv Res. 2024 Nov 29;24:1509. doi: 10.1186/s12913-024-11965-2 (PMC11606115; doi:10.1186/s12913-024-11965-2)
Supplement: Supplementary file 1 — Supplementary Material 1. [file 12913_2024_11965_MOESM1_ESM.docx]

Appendix A: Search strategies

Ovid MEDLINE

[https://ovidsp.ovid.com/ovidweb.cgi?T=JS&NEWS=N&PAGE=main&SHAREDSEARCHID=15u3KpvAecZ6F4B9sNdiosRXMpnjYHwT8cKPPuTFNXNgnvlbo27FOquHyBEAPQIj#](https://ovidsp.ovid.com/ovidweb.cgi?T=JS&NEWS=N&PAGE=main&SHAREDSEARCHID=15u3KpvAecZ6F4B9sNdiosRXMpnjYHwT8cKPPuTFNXNgnvlbo27FOquHyBEAPQIj)

**Medline** [Ovid MEDLINE® Epub Ahead of Print, In-Process & Other Non-Indexed Citations, Ovid MEDLINE® Daily and Ovid MEDLINE®] 1946 to present

1 ["physician* associate*" or "physician* assistant*" or "medical associate profession*" or "advanced practice provider*" or "mid-level provider*" or "mid-level practitioner*"].ti,ab. 7237

2 exp Physician Assistants/ 6555

3 1 or 2 10860

4 [UK or "United Kingdom*" or England* or English or Scotland* or Scottish or Wales or Welsh or Ireland* or Irish or Britain* or GB or British or NHS or "National Health Service"].ti,ab. 611325

5 exp United Kingdom/ 393718

6 National Health Programs/ 33706

7 4 or 5 or 6 872161

8 [patient* or public or experience* or perspective* or perception* or perceive* or satisfaction or contribution or willingness or evaluation or attitude or view* or beliefs or accept* or opinion* or knowledge or understand* or aware* or recognition or recognise*].ti,ab. 13508832

9 exp Patient Satisfaction/ 100705

10 "Document Analysis"/ or focus groups/ or interviews as topic/ or narration/ or qualitative research/ or [[depth or face or group or guided or indepth or informal or semistructured or structured or unstructured] adj4 [discussion or discussions or interview or interviewed or interviews or survey or surveys]].ti,ab,kf. or [ethnographic or ethnography or [field adj1 work] or fieldwork or [focus adj1 [group or groups]] or [groups adj2 interviewed] or [key adj1 [informant or informants]] or [qualitative adj2 [research or studies or studies]]].ti,ab,kf. 381146

11 8 or 9 or 10 13581505

12 3 and 7 and 11 337

13 limit 12 to yr="2003 -Current" 276 10/04

Ovid EMBASE

<https://ovidsp.ovid.com/ovidweb.cgi?T=JS&NEWS=N&PAGE=main&SHAREDSEARCHID=bC5mE25Uxy5whjE7VgcrCIkwQNvApeXzq8GgMTaAPVUcsmBRwEpURNrNuyXEvdBV>

**Embase** 1974 to present

1 ["physician* associate*" or "physician* assistant*" or "medical associate profession*" or "advanced practice provider*" or "mid-level provider*" or "mid-level practitioner*"].ti,ab. 10725

2 physician assistant/ 9731

3 1 or 2 15037

4 [UK or "United Kingdom*" or England* or English or Scotland* or Scottish or Wales or Welsh or Ireland* or Irish or Britain* or GB or British or NHS or "National Health Service"].ti,ab. 971200

5 exp United Kingdom/ 473561

6 national health service/ 73572

7 4 or 5 or 6 1202615

8 [patient* or public or experience* or perspective* or perception* or perceive* or satisfaction or contribution or willingness or evaluation or attitude or view* or beliefs or accept* or opinion* or knowledge or understand* or aware* or recognition or recognise*].ti,ab. 18103202

9 patient satisfaction/ 175764

10 exp interview/ or narrative/ or exp qualitative research/ or [[depth or face or group or guided or indepth or informal or semistructured or structured or unstructured] adj4 [discussion or discussions or interview or interviewed or interviews or survey or surveys]].ti,ab,kf. or [ethnographic or ethnography or [field adj1 work] or fieldwork or [focus adj1 [group or groups]] or [groups adj2 interviewed] or [key adj1 [informant or informants]] or [qualitative adj2 [research or studies or studies]]].ti,ab,kf. 670836

11 8 or 9 or 10 18262130

12 3 and 7 and 11 441

13 limit 12 to yr="2003 -Current" 414 10/04

Ovid PsycINFO

<https://ovidsp.ovid.com/ovidweb.cgi?T=JS&NEWS=N&PAGE=main&SHAREDSEARCHID=1TVlPxHAjwpKY87PU2SJcDgScQet8bSU1iJNCtbtKzPyXEfL8qPfpSaUb6ujLHU1e>

**PsycINFO** 1806 to present

1 ["physician* associate*" or "physician* assistant*" or "medical associate profession*" or "advanced practice provider*" or "mid-level provider*" or "mid-level practitioner*"].ti,ab. 1051

2 [UK or "United Kingdom*" or England* or English or Scotland* or Scottish or Wales or Welsh or Ireland* or Irish or Britain* or GB or British or NHS or "National Health Service"].ti,ab. 272290

3 [patient* or public or experience* or perspective* or perception* or perceive* or satisfaction or contribution or willingness or evaluation or attitude or view* or beliefs or accept* or opinion* or knowledge or understand* or aware* or recognition or recognise*].ti,ab. 3199104

4 patient satisfaction/ 6685

5 exp qualitative methods/ 22021

6 [[[depth or face or group or guided or indepth or informal or semistructured or structured or unstructured] adj4 [discussion or discussions or interview or interviewed or interviews or survey or surveys]] or [ethnographic or ethnography or [field adj1 work] or fieldwork or [focus adj1 [group or groups]] or [groups adj2 interviewed] or [key adj1 [informant or informants]] or [qualitative adj2 [research or studies or studies]]]].ti,ab. 252235

7 3 or 4 or 5 or 6 3242405

8 1 and 2 and 7 24

9 8 24

10 limit 9 to yr="2003 -Current" 24 10/04/2024

EBSCOhost CINAHL

| **CINAHL** 10/04/2024 | Wednesday, April 10, 2024 2:51:04 PM |
| --- | --- |

| **#** | **Query** | | | **Results** |
| --- | --- | --- | --- | --- |
| S1 | [MH "Physician Assistants"] | | | 5,706 |
| S2 | TI [ ["physician* associate*" or "physician* assistant*" or "medical associate profession*" or "advanced practice provider*" or "mid-level provider*" or "mid-level practitioner*"] ] OR AB [ ["physician* associate*" or "physician* assistant*" or "medical associate profession*" or "advanced practice provider*" or "mid-level provider*" or "mid-level practitioner*"] ] | | | 5,176 |
| S3 | S1 OR S2 | | | 8,764 |
| S4 | [MH "United Kingdom+"] | | | 324,879 |
| S5 | [MH "National Health Programs"] | | | 68,054 |
| S6 | TI [ UK or "United Kingdom*" or England* or English or Scotland* or Scottish or Wales or Welsh or Ireland* or Irish or Britain* or GB or British or NHS or "National Health Service" ] OR AB [ UK or "United Kingdom*" or England* or English or Scotland* or Scottish or Wales or Welsh or Ireland* or Irish or Britain* or GB or British or NHS or "National Health Service" ] | | | 250,464 |
| S7 | S4 OR S5 OR S6 | | | 492,441 |
| S8 | TI [ [patient* or public or experience* or perspective* or perception* or perceive* or satisfaction or contribution or willingness or evaluation or attitude or view* or beliefs or accept* or opinion* or knowledge or understand* or aware* or recognition or recognise* ] OR AB [ [patient* or public or experience* or perspective* or perception* or perceive* or satisfaction or contribution or willingness or evaluation or attitude or view* or beliefs or accept* or opinion* or knowledge or understand* or aware* or recognition or recognise* ] | | | 3,382,001 |
| S9 | [MH "Patient Satisfaction+"] | | | 65,697 |
| S10 | [MH "Qualitative Studies+"] | | | 192,946 |
| S11 | | [MH "Focus Groups"] OR [MH "Interviews+"] OR [MH "Narratives+"] | | 305,131 |
| S12 | TI [ [[[depth or face or group or guided or indepth or informal or semistructured or structured or unstructured] n4 [discussion or discussions or interview or interviewed or interviews or survey or surveys]] or [ethnographic or ethnography or [field n1 work] or fieldwork or [focus n1 [group or groups]] or [groups n2 interviewed] or [key n1 [informant or informants]] or [qualitative n2 [research or studies or studies]]]] ] OR AB [ [[[depth or face or group or guided or indepth or informal or semistructured or structured or unstructured] n4 [discussion or discussions or interview or interviewed or interviews or survey or surveys]] or [ethnographic or ethnography or [field n1 work] or fieldwork or [focus n1 [group or groups]] or [groups n2 interviewed] or [key n1 [informant or informants]] or [qualitative n2 [research or studies or studies]]]] ] | |  |  |
| S13 | S8 OR S9 OR S10 OR S11 OR S12 | | | 3,501,101 |
| S14 | S3 AND S7 AND S13 | | | 170 |
| S15 | S3 AND S7 AND S13 **Limiters** - Publication Date: 20030101-20241231 | | | 161 10/04 |

Bottom of Form

Education Resources Information Centre

S1
title["physician* associate*" or "physician* assistant*" or "medical associate profession*" or "advanced practice provider*" or "mid-level provider*" or "mid-level practitioner*"] OR abstract["physician* associate*" or "physician* assistant*" or "medical associate profession*" or "advanced practice provider*" or "mid-level provider*" or "mid-level practitioner*"]

S2
title[UK or "United Kingdom*" or England* or English or Scotland* or Scottish or Wales or Welsh or Ireland* or Irish or Britain* or GB or British or NHS or "National Health Service"] OR abstract[UK or "United Kingdom*" or England* or English or Scotland* or Scottish or Wales or Welsh or Ireland* or Irish or Britain* or GB or British or NHS or "National Health Service"]

S3
abstract[patient* or public or experience* or perspective* or perception* or perceive* or satisfaction or contribution or willingness or evaluation or attitude or view* or beliefs or accept* or opinion* or knowledge or understand* or aware* or recognition or recognise*] OR title[patient* or public or experience* or perspective* or perception* or perceive* or satisfaction or contribution or willingness or evaluation or attitude or view* or beliefs or accept* or opinion* or knowledge or understand* or aware* or recognition or recognise*]

S4

[abstract[[[[depth or face or group or guided or indepth or informal or semistructured or structured or unstructured] n/4 [discussion or discussions or interview or interviewed or interviews or survey or surveys]] or [ethnographic or ethnography or [field n/1 work] or fieldwork or [focus n/1 [group or groups]] or [groups n/2 interviewed] or [key n/1 [informant or informants]] or [qualitative n/2 [research or studies or studies]]]]] OR title[[[[depth or face or group or guided or indepth or informal or semistructured or structured or unstructured] n/4 [discussion or discussions or interview or interviewed or interviews or survey or surveys]] or [ethnographic or ethnography or [field n/1 work] or fieldwork or [focus n/1 [group or groups]] or [groups n/2 interviewed] or [key n/1 [informant or informants]] or [qualitative n/2 [research or studies or studies]]]]]](https://www.proquest.com/recentsearches.recentsearchtabview.recentsearchesgridview.scrolledrecentsearchlist.checkdbssearchlink:rerunsearch/EB0D8FCE521D4234PQ/None/$N?site=eric&t:ac=RecentSearches)

S5

[MAINSUBJECT.EXACT.EXPLODE["Qualitative Research"]](https://www.proquest.com/recentsearches.recentsearchtabview.recentsearchesgridview.scrolledrecentsearchlist.checkdbssearchlink:rerunsearch/1F81440612CE4000PQ/None/$N?site=eric&t:ac=RecentSearches)

S6 S3 OR S4 OR S5

S7 S1 AND S2 AND S6

S8 S1 AND S2 AND S6: 2003-2024  7 total 10/04/2024

**ProQuest Dissertations and Theses Global**

S1
title["physician* associate*" or "physician* assistant*" or "medical associate profession*" or "advanced practice provider*" or "mid-level provider*" or "mid-level practitioner*"] OR abstract["physician* associate*" or "physician* assistant*" or "medical associate profession*" or "advanced practice provider*" or "mid-level provider*" or "mid-level practitioner*"]

S2

title[UK or "United Kingdom*" or England* or English or Scotland* or Scottish or Wales or Welsh or Ireland* or Irish or Britain* or GB or British or NHS or "National Health Service"] OR abstract[UK or "United Kingdom*" or England* or English or Scotland* or Scottish or Wales or Welsh or Ireland* or Irish or Britain* or GB or British or NHS or "National Health Service"]

S3

abstract[patient* or public or experience* or perspective* or perception* or perceive* or satisfaction or contribution or willingness or evaluation or attitude or view* or beliefs or accept* or opinion* or knowledge or understand* or aware* or recognition or recognise*] OR title[patient* or public or experience* or perspective* or perception* or perceive* or satisfaction or contribution or willingness or evaluation or attitude or view* or beliefs or accept* or opinion* or knowledge or understand* or aware* or recognition or recognise*]

S4

[abstract[[[[depth or face or group or guided or indepth or informal or semistructured or structured or unstructured] n/4 [discussion or discussions or interview or interviewed or interviews or survey or surveys]] or [ethnographic or ethnography or [field n/1 work] or fieldwork or [focus n/1 [group or groups]] or [groups n/2 interviewed] or [key n/1 [informant or informants]] or [qualitative n/2 [research or studies or studies]]]]] OR title[[[[depth or face or group or guided or indepth or informal or semistructured or structured or unstructured] n/4 [discussion or discussions or interview or interviewed or interviews or survey or surveys]] or [ethnographic or ethnography or [field n/1 work] or fieldwork or [focus n/1 [group or groups]] or [groups n/2 interviewed] or [key n/1 [informant or informants]] or [qualitative n/2 [research or studies or studies]]]]]](https://www.proquest.com/recentsearches.recentsearchtabview.recentsearchesgridview.scrolledrecentsearchlist.checkdbssearchlink:rerunsearch/EB0D8FCE521D4234PQ/None/$N?site=eric&t:ac=RecentSearches)

S5 S3 OR S4

S6 S1 AND S2 AND S5

S7 S1 AND S2 AND S5: 2003-2024  14 results 10/04/2024

Scopus

[ TITLE-ABS-KEY [ [ "physician* associate*" OR "physician* assistant*" OR "medical associate profession*" OR "advanced practice provider*" OR "mid-level provider*" OR "mid-level practitioner*" ] ] ] AND [ TITLE-ABS-KEY [ [ uk OR "United Kingdom*" OR england* OR english OR scotland* OR scottish OR wales OR welsh OR ireland* OR irish OR britain* OR gb OR british OR nhs OR "National Health Service" ] ] ] AND [ [ TITLE-ABS-KEY [ [ patient* OR public OR experience* OR perspective* OR perception* OR perceive* OR satisfaction OR contribution OR willingness OR evaluation OR attitude OR view* OR beliefs OR accept* OR opinion* OR knowledge OR understand* OR aware* OR recognition OR recognise* ] ] ] OR [ TITLE-ABS-KEY [ [ [ [ [ depth OR face OR group OR guided OR indepth OR informal OR semistructured OR structured OR unstructured ] W/4 [ discussion OR discussions OR interview OR interviewed OR interviews OR survey OR surveys ] ] OR [ ethnographic OR ethnography OR [ field W/1 work ] OR fieldwork OR [ focus W/1 [ group OR groups ] ] OR [ groups W/2 interviewed ] OR [ key W/1 [ informant OR informants ] ] OR [ qualitative W/2 [ research OR studies OR studies ] ] ] ] ] ] ] ] AND PUBYEAR > 2002 AND PUBYEAR < 2025 160 10/04
