# Supplementary material for: Public perception of the physician associate profession in the UK: a systematic review
Source: BMC Health Serv Res. 2024 Nov 29;24:1509. doi: 10.1186/s12913-024-11965-2 (PMC11606115; doi:10.1186/s12913-024-11965-2)
Supplement: Supplementary file 2 — Supplementary Material 2. [file 12913_2024_11965_MOESM2_ESM.docx]

| CASP Quality Appraisal Checklists | | | | | | | | | | | | |
| --- | --- | --- | --- | --- | --- | --- | --- | --- | --- | --- | --- | --- |
| Papers | **Q. 1** | **Q. 2** | **Q. 3** | **Q. 4** | **Q. 5** | **Q. 6** | **Q. 7** | **Q. 8** | **Q. 9** | **Q. 10** | **Quality Appraisal** | **Overview** |
| Jackson et al [2017] | 2 | 2 | 2 | 2 | 2 | 1 | 0 | 2 | 2 | 2 | Moderate | Data collection method was stated clearly [focus groups] and is appropriate for the study. While the researchers do not explicitly justify the focus groups, they are an effective means of collecting qualitative data. No method for conducting the focus group interviews was included but the analysis method of made clear. The researchers state that they have a conflicting interest, but did not critically examine any potential biases in the text. No mention of ethical concerns was made. A detailed description of the analysis process was included and the themes are stated clearly in the results section. The themes are stated clearly in the results section. |
| Shah et al [2021] | 2 | 2 | 1 | 2 | 2 | 0 | 0 | 1 | 2 | 2 | Low | Little depth of analysis for relevant data. Ethical issues not considered, and little information on participants or recruitment present. |
| Taylor et al [2020] | 2 | 2 | 2 | 2 | 2 | 2 | 2 | 2 | 2 | 2 | High | Methodology justified well in the study - focus was on understanding patient experience. Study design justified in the paper. Participants were patients who were asked to partake by who was involved in the project. Though there may be bias, this is an efficient way of recruiting participants. Paper made no mention of researcher bias involved. They did however note that there was a possibility of a selection bias. |
| Taylor et al [2019] | 2 | 2 | 2 | 2 | 2 | 2 | 2 | 2 | 2 | 2 | High | Study defines goals clearly Important as it addresses gap in the literature. Study defines goals clearly Important as it addresses gap in the literature. Findings are described in detail and clearly stated, and steps were taken to minimise bias. |
| McDermott et al [2022] | 2 | 2 | 2 | 2 | 2 | 2 | 2 | 2 | 2 | 2 | High | Little to no mention of potential researcher bias but otherwise very sound. Study design is appropriate [mixed-methods which each tackle different objectives] and participant recruitment and data collection methods clear and justified. |
| Farmer et al [2011] | 2 | 2 | 2 | 2 | 1 | 0 | 2 | 1 | 2 | 2 | Moderate | Qualitative design is appropriate as the study is evaluation impact of PAs; this would be difficult to measure quantitively. Some detail given on recruitment but not much. Researcher bias not considered and data analysis not described in detail, making it difficult to determine its quality. |
| Williams & Ritsema [2017] | 2 | 2 | 2 | 2 | 2 | 2 | 2 | 1 | 2 | 2 | High | Only descriptive data collected; little data analysis presented. Biases taken into consideration. Methods and results clear, detailed, and justified. |
| Halter et al [2020] | 2 | 2 | 2 | 2 | 2 | 2 | 2 | 2 | 2 | 2 | High | Much detail given regarding participants and recruitment, and methodology. Little relevant data present, however. |
| Halter et al [2017c] | 2 | 2 | 2 | 2 | 2 | 2 | 2 | 2 | 2 | 2 | High | Researcher bias not considered. Consent given, does not explicitly state it was informed consent. Ethical approval was sought. Findings fill gap in the literature. |
| Cheang et al [2009] | 2 | 2 | 1 | 1 | 1 | 0 | 0 | 1 | 2 | 1 | Low | Quantitative method is somewhat appropriate. No discussion as to the justifications behind the research methods present and little info on participants given. Researcher bias not considered. Participants anonymised. No strong ethical issues present. No analytic details given. |
| Zaman et al [2018b] | 2 | 2 | 2 | 2 | 2 | 0 | 1 | 2 | 2 | 1 | High | The ethical approval committee deemed this study exempt from ethical approval. Does not state if informed consent was taken or how. Study is otherwise methodologically sound. |
| Drennan et al [2020] | 2 | 2 | 2 | 2 | 2 | 0 | 2 | 2 | 2 | 2 | High | Researcher bias not considered. Criticism of findings discussed in some detail. |
| Drennan et al [2019] | 2 | 2 | 2 | 2 | 2 | 2 | 2 | 1 | 2 | 1 | High | Study acknowledges potential researcher bias and flaws. Study is methodologically sound and results and clear and detailed. |
| Drennan et al [2019] [PA-SCER] | 2 | 2 | 2 | 2 | 2 | 2 | 2 | 2 | 2 | 2 | High | Detailed recruitment strategy: study conducted preliminary recruitment first. Methodology is detailed and clear. Study discusses steps it took to minimise bias. Study results are discussed in detail. Ethical approval was sought and received. |
| Drennan et al [2015] | 2 | 2 | 2 | 2 | 2 | 0 | 2 | 2 | 2 | 2 | High | Took steps to minimise bias including all records as part of the study. Study received ethical approval, does not explicitly state whether informed consent was taken. Analysis described in detail. |
| Drennan et al [2014] | 2 | 2 | 2 | 2 | 2 | 2 | 2 | 2 | 2 | 2 | High | Methodology is appropriate for the study’s intentions and the research design accurately addresses the research questions: it is mixed-methods. Took steps to minimise bias including all records as part of the study. |
| Woodin et al [2005] | 2 | 2 | 2 | 1 | 1 | 0 | 2 | 1 | 2 | 2 | Moderate | Researcher bias was considered: data were coded blind and coded separately when more than one presenting problem was recorded. Study contains some information on the analysis but could have contained more so that a more accurate quality appraisal could be made. |
| Wilsher et al [2023] | 2 | 2 | 2 | 1 | 2 | 2 | 2 | 2 | 2 | 2 | High | Study is detailed with a clear methods and results section. Researcher bias taken into account and analysed described as well. |

Appendix B: CASP quality appraisal checklist

Key: 0 = No; 1 = Can’t Tell; 2 = Yes.
