# Supplementary material for: Public perception of the physician associate profession in the UK: a systematic review
Source: BMC Health Serv Res. 2024 Nov 29;24:1509. doi: 10.1186/s12913-024-11965-2 (PMC11606115; doi:10.1186/s12913-024-11965-2)
Supplement: Supplementary file 3 — Supplementary Material 3. [file 12913_2024_11965_MOESM3_ESM.docx]

| CERQual Assessment | | | | | | | |
| --- | --- | --- | --- | --- | --- | --- | --- |
| Review Finding | **Studies Contributing to the Review Finding** | **Assessment of the Methodological Limitations** | **Assessment of Relevance** | **Assessment of Coherence** | **Assessment of Adequacy** | **Overall CERQual Assessment of Confidence** | **Explanation of Judgement** |
| Patients lacking information about PAs | Studies 1; 3; 4; 7; 9; 10; 13; 14; 16; 17, 18 | Few methodological limitations [1 study with minor issues, 1 study with moderate issues] | Minor concerns about relevance [studies of both primary and secondary care with mostly patient participants] | Minor concerns about data coherence [data consistent across the studies] | Minor concerns about adequacy [five studies rich in detail, 1 study with moderate detail] | High confidence | Few concerns about methodological limitations, relevance, and adequacy of data. Data is rich in detail and analysis. |
| PAs mistaken for other healthcare workers | Studies 3; 4; 7; 8; 9; 13; 14; 16; 17, 18. | Few methodological limitations [1 study with minor issues] | Minor concerns about relevance [studies of both primary and secondary care with mostly patient participants] | Minor concerns about data coherence [data consistent across the studies] | Moderate concerns about adequacy [five studies with moderate detail, some quantitative data from other studies supporting results however] | Moderate confidence | Some concern about adequacy of data [Many studies contain relevant data but only small amounts]. |
| Patients’ experiences being seen by a PA | Studies 1; 2; 3; 4; 5; 6; 7; 9; 11; 12; 13; 14; 15; 16; 17, 18. | Few methodological limitations [2 studies with minor issues, 1 study with moderate issues] | Minor concerns about relevance [studies of both primary and secondary care with mostly patient participants] | Minor concerns about data coherence [data consistent across the studies] | Minor concerns about adequacy [7 studies rich in detail, 2 studies with moderate detail] | High confidence | Few concerns about methodological limitations, relevance, and adequacy of data. Data is rich in detail and analysis. |
| Patient willingness to be seen by a PA | Studies 3; 4; 5; 7; 9; 11; 12; 13; 14; 16; 17, 18. | Few methodological limitations [1 study with minor issues] | Minor concerns about relevance [studies of both primary and secondary care with mostly patient participants] | Minor concerns about data coherence [data consistent across the studies] | Moderate concerns about adequacy [1 study rich in detail, 3 studies with moderate data] | Moderate confidence | Some concern about adequacy of data [Many studies contain relevant data but only small amounts]. |
| Patient perception of the healthcare system | Studies 4; 8; 9; 11; 12; 14; 15; 16; 17. | Few methodological limitations [1 study with minor issues] | Minor concerns about relevance [studies of both primary and secondary care with mostly patient participants] | Some concerns about data coherence [data given in small quantities across studies and not always analysed in depth] | Substantial concerns about adequacy [3 studies with moderately rich data, thin data from the remaining 6 studies] | Low confidence | Concerns about adequacy of data due to small amount of data collected across the studies]. |

Appendix C: Grade-CERQual assessment
